# Nitroglycerin reverts clinical manifestations of poor peripheral perfusion in patients with circulatory shock

**DOI:** 10.1186/cc13932

**Published:** 2014-06-19

**Authors:** Alexandre Lima, Michel E van Genderen, Jasper van Bommel, Eva Klijn, Tim Jansem, Jan Bakker

**Affiliations:** 1 Department of Intensive Care, Erasmus MC University Hospital Rotterdam, PO Box 2040, 3000, CA Rotterdam, The Netherlands

## Abstract

**Introduction:**

Recent clinical studies have shown a relationship between abnormalities in peripheral perfusion and unfavorable outcome in patients with circulatory shock. Nitroglycerin is effective in restoring alterations in microcirculatory blood flow. The aim of this study was to investigate whether nitroglycerin could correct the parameters of abnormal peripheral circulation in resuscitated circulatory shock patients.

**Methods:**

This interventional study recruited patients who had circulatory shock and who persisted with abnormal peripheral perfusion despite normalization of global hemodynamic parameters. Nitroglycerin started at 2 mg/hour and doubled stepwise (4, 8, and 16 mg/hour) each 15 minutes until an improvement in peripheral perfusion was observed. Peripheral circulation parameters included capillary refill time (CRT), skin-temperature gradient (Tskin-diff), perfusion index (PI), and tissue oxygen saturation (StO_2_) during a reactive hyperemia test (RincStO_2_). Measurements were performed before, at the maximum dose, and after cessation of nitroglycerin infusion. Data were analyzed by using linear model for repeated measurements and are presented as mean (standard error).

**Results:**

Of the 15 patients included, four patients (27%) responded with an initial nitroglycerin dose of 2 mg/hour. In all patients, nitroglycerin infusion resulted in significant changes in CRT, Tskin-diff, and PI toward normal at the maximum dose of nitroglycerin: from 9.4 (0.6) seconds to 4.8 (0.3) seconds (*P* <0.05), from 3.3°C (0.7°C) to 0.7°C (0.6°C) (*P* <0.05), and from [log] -0.5% (0.2%) to 0.7% (0.1%) (*P* <0.05), respectively. Similar changes in StO_2_ and RincStO_2_ were observed: from 75% (3.4%) to 84% (2.7%) (*P* <0.05) and 1.9%/second (0.08%/second) to 2.8%/second (0.05%/second) (*P* <0.05), respectively. The magnitude of changes in StO_2_ was more pronounced for StO_2_ of less than 75%: 11% versus 4%, respectively (*P* <0.05).

**Conclusions:**

Dose-dependent infusion of nitroglycerin reverted abnormal peripheral perfusion and poor tissue oxygenation in patients following circulatory shock resuscitation. Individual requirements of nitroglycerin dose to improve peripheral circulation vary between patients. A simple and fast physical examination of peripheral circulation at the bedside can be used to titrate nitroglycerin infusion.

## Introduction

In the early 1960s, the use of vasodilators in shock started with the interest in flow more than in pressure [1]. This was followed by clinical observations and some experimental studies showing the beneficial effects of vasodilators in severe (irreversible) shock [2,3]. It took many years before this topic was the subject of additional experimental studies including new techniques to monitor the effects of vasodilators [4-9]. These findings inspired some clinical investigators to propose the administration of nitroglycerin as a therapeutic approach to recruit the (sublingual) microcirculation in septic shock and cardiogenic shock [10-12]. Although these studies showed that nitroglycerin is effective in restoring abnormal sublingual microcirculation, the implementation of such a therapeutic approach in clinical practice is still hampered by technical aspects and complex offline analysis of the images.

A real-time evaluation of peripheral microcirculatory disorders would provide bedside assessment for timely application of nitroglycerin targeting improvement in microcirculatory perfusion. In addition, recent observations have demonstrated a significant association between the persistence of abnormalities in peripheral circulation, measured in skin, muscle, or sublingual mucosa, with more severe organ dysfunction and worse prognosis when compared with traditional global variables of resuscitation [13-21]. Although these abnormalities in peripheral perfusion predict unfavorable outcome in critically ill patients, it still needs to be proven that these abnormalities not only can be treated but also result in improved morbidity or mortality or both. It is reasonable, therefore, that these fundamental questions ideally be answered before the introduction of a new monitoring parameter in clinical practice [22].

Monitoring of peripheral perfusion can be performed by using simple clinical assessment, in particular, the physical examination by touching the skin or measuring capillary refill time (CRT), body temperature gradient, and optical devices, such as pulse oximeter signal and tissue oxygen saturation (StO_2_) [23]. We question whether these easy, reliable, and robust clinical parameters of peripheral perfusion can be an effective monitoring approach at the bedside to titrate the beneficial effects of nitroglycerin. Therefore, we designed the present study to address two general hypotheses. First, can nitroglycerin revert abnormal peripheral circulation that persists after initial resuscitation in patients with circulatory shock? Second, is there a dose-dependent effect that would require individualization of the nitroglycerin dose?

## Materials and methods

### Setting and participants

This was an intervention study within the intensive care unit (ICU) of a university hospital. The local accredited Medical Research Ethical Committee from Erasmus MC Hospital approved the study. Written informed consent was obtained from all patients, their next of kin, or a surrogate decision maker, as appropriate. All consecutive patients admitted in the ICU for circulatory shock resuscitation were eligible for inclusion. Circulatory shock was defined as hypotension (systolic blood pressure of less than 90 mm Hg, mean arterial pressure of less than 70 mm Hg, or a systolic blood pressure decrease of greater than 40 mm Hg below normal range), despite adequate fluid resuscitation or the requirement for continuous norepinephrine infusion, and the presence of metabolic acidosis (arterial pH of less than 7.35 or base excess of less than -3 mmol/L) in association with increased lactate levels (>2 mmol/L). Patients were eligible for inclusion if, after 6 hours of ICU admission and continuous resuscitation and stabilization, abnormal peripheral perfusion was still present despite normalization of global hemodynamic parameters. Accordingly, all patients included shared abnormalities in peripheral perfusion as baseline characteristics. Exclusion criteria were arm injury or ischemia from trauma (disturbing measurement of peripheral circulation), liver failure, and any neurological insult that could lead to increased intracranial pressure (stroke, subarachnoid hemorrhage, or brain trauma injury).

### Measurements

All patients were monitored with a radial artery catheter for continuous arterial pressure monitoring. Global hemodynamic variables included heart rate, central venous pressure, and mean arterial pressure. All measurements were obtained by using standard equipment. Cardiac output was obtained if the patient was monitored with continuous pulse contour cardiac analysis (PiCCO; Pulsion Medical Systems AG, Munich, Germany).

Peripheral circulation parameters included physical examination of peripheral perfusion with CRT, forearm-to-fingertip skin-temperature gradient (Tskin-diff), and peripheral perfusion index (PI) from pulse oximeter signal. CRT was measured by applying firm pressure to the distal phalanx of the index finger for 15 seconds. A chronometer recorded the time for the return of normal color, and 5 seconds was defined as the upper limit of normality [24]. Tskin-diff was obtained with two skin probes (Hewlett-Packard 21078A; Hewlett-Packard, Palo Alto, CA, USA) attached to the index finger and on the radial side of the forearm, midway between the elbow and the wrist. Tskin-diff can better reflect changes in cutaneous blood flow than skin temperature itself. When evaluated under constant environmental conditions, Tskin-diff increases during vasoconstriction, and a threshold of 2°C has been shown to reflect vasoconstriction in critically ill patients [25,26]. Peripheral PI provides a non-invasive method for evaluating perfusion and has been shown to reflect changes in peripheral circulation [27]. In this study, the peripheral PI value was obtained by using a pulse oximeter (Masimo® SET Radical-7; Masimo Corporation, Irvine, CA, USA), which displays a range from 0.02% (very weak pulse strength) to 20.0% (very strong pulse strength). In addition, we used near-infrared spectroscopy for StO_2_-derived tissue oxygenation measurements to investigate the dynamic changes between tissue oxygenation and condition of peripheral perfusion. StO_2_-derived tissue oxygenation was monitored continuously by using an InSpectra Tissue Spectrometer Model 650 (Hutchinson Technology Inc., Hutchinson, MN, USA) with a 15-mm probe over the thenar eminence. A vascular occlusion test was performed by arrest of forearm blood flow by using a conventional sphygmomanometer pneumatic cuff. The cuff was placed around the upper arm and was inflated to a pressure approximately 30 mm Hg greater than patient systolic pressure for 3 minutes. Upon completion of the ischemic period, the occluding cuff was rapidly deflated to 0 mm Hg. The derived StO_2_ parameters were divided into three components: resting StO_2_ values, rate of StO_2_ desaturation (RdecStO_2_, expressed as percentage per minute), and rate of StO_2_ recovery (RincStO_2_, expressed as percentage per second).

Patients were considered to have abnormal peripheral circulation if the examined extremities (both hands) had an increase in CRT of greater than 5 seconds or indicated the presence of peripheral vasoconstriction (Tskin-diff of greater than 2°C and peripheral PI of less than 1.4%). The ICU has single-patient closed rooms, and the ambient temperature in each room was individually and actively set at 22°C.

### Study design

**Figure 1:**
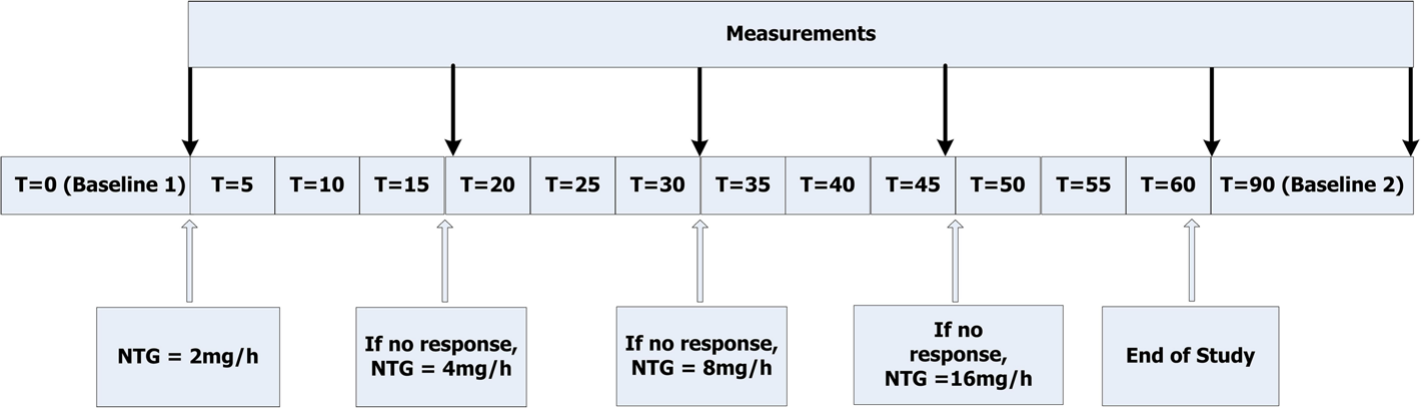
**Flowchart of the study protocol.** Time points of the study were defined as baseline 1 before nitroglycerin infusion (T_BL1_), time point when peripheral perfusion was normalized at the maximum dose of nitroglycerin (T_MX_), and baseline 2 recorded 30 minutes after cessation of nitroglycerin infusion (T_BL2_). NTG, nitroglycerin.

To avoid significant hypotension during nitroglycerin infusion, each patient was evaluated for adequate intravascular volume as evidenced by repeated volume challenges (250 mL of crystalloid over 10 minutes) up to a point at which central venous pressure raised by more than 2 mm Hg or stroke volume did not increase more than 10%. After documentation of central venous pressure or stroke volume changes, a set of measurements was obtained during a control period. After baseline measurements, nitroglycerin was given as a bolus equal to the volume of the used infusion line followed by a continuous intravenous infusion initiated at 2 mg/hour (33.3 μg/minute). When peripheral circulation did not normalize within 15 minutes after the start of the nitroglycerin infusion, repeated measurements of hemodynamic and peripheral perfusion parameters were recorded and the dose was doubled. This was repeated until peripheral perfusion was normalized or a dose of 16 mg/hour was reached (Figure 1). The stepwise increase in nitroglycerin infusion rate resulted in the following dosages used: 4 mg/hour (66.6 μg/minute), 8 mg/hour (133.3 μg/minute), and 16 mg/hour (266.6 μg/minute). We defined improvement in peripheral perfusion as a change of more than 50% in baseline parameters of CRT or the presence of peripheral vasodilation (Tskin-diff of less than 2°C or peripheral PI of greater than 1.4%). At the end of the stepwise increase in nitroglycerin when peripheral circulation was corrected and a final set of measurements had been made, the infusion of nitroglycerin was stopped. A second set of baseline measurements was recorded 30 minutes after cessation of the infusion (Figure 1).

The nitroglycerin infusion was stopped if the patient developed significant hypotension (mean arterial pressure of less than 50 mm Hg). During the study, infusion rates of noradrenaline or other vasoactive drugs were not changed and no additional fluids were administered.

### Statistical analysis

Unless otherwise specified, descriptive analyses are reported as median (25th-75th). Time points of the study were defined as T_BL1_ (baseline before nitroglycerin infusion), T_MX_ (at the maximum dose of nitroglycerin), and T_BL2_ (30 minutes after cessation of nitroglycerin infusion). We used a Kolmogorov-Smirnov test and stratified distribution plots to verify the normality of distribution of continuous variables. Not normally distributed data underwent log transformation to achieve close to normal distribution and then qualified for longitudinal testing. We used linear model for repeated measurements (time points as independent factor) to investigate changes in the average of hemodynamic and peripheral perfusion parameters (dependent variables). The analyses of linear model are reported as mean response (standard error). Differences between groups’ means were tested by Mann-Whitney *U* test. A multiple regression analysis was applied to estimate the effect of nitroglycerin dose (independent variable) on parameters of peripheral perfusion (dependent variables) so that we could predict changes in CRT, Tskin-diff, peripheral PI, and StO_2_ for a given nitroglycerin dose. SPSS (version 15.0; SPSS, Inc., Chicago, IL, USA) was used for statistical analysis. A *P* value of less than 0.05 was considered statistically significant.

## Results

Of the 15 patients included in the study, 12 had septic shock and three had non-septic shock. Patients’ demographic data are summarized in Table 1. At the moment of inclusion (6 hours after ICU admission), all patients had central venous oxygen saturation of at least 70% and four patients had hyperlactatemia (lactate of greater than 2.0 mmol/L). The total amount of volume for fluid challenge necessary for each patient was 416 ± 204 mL. As the protocol was based on dose response, some patients had longer durations of nitroglycerin infusion than others. In only four patients (27%), nitroglycerin infusion response was observed with the initial dose of 2 mg/hour. In two patients, the highest dose of nitroglycerin (16 mg/hour) was necessary to improve peripheral perfusion; five patients required 4 mg/hour, and four patients required 8 mg/hour. Table 2 shows the hemodynamic effects of nitroglycerin infusion during execution of the study protocol stratified by the time points. In all patients, mean arterial pressure was significantly lower at the maximum-dose time point (T_MX_). Cardiac index and stroke volume were measured in six patients; although both parameters showed a slight decrease, no significant differences were observed during nitroglycerin infusion.

**Table 1:** Baseline characteristics of the patients

**Patient demographic data**	
Number of patients	15
Age, years	63 (48-71)
Male/Female	9/6
SOFA score	10 (5-11)
APACHE II score	22 (16-27)
Admission category:	6 abdominal sepsis
	5 pneumonia
	2 postoperative
	1 hemorrhagic shock
	1 meningitis
Noradrenaline use, number (percentage)	14 (93%)
Noradrenaline dose, μg/kg per minute	0.13 (0.03-0.40)
Mechanical ventilation, number (percentage)	15 (100%)
Lactate, mmol/L	1.8 (1.1-2.1)
Survivor/Non-survivor	10/5

Values are expressed as median (25th-75th) except where indicated otherwise. APACHE II, Acute Physiology and Chronic Health Evaluation II; SOFA, Sequential Organ Failure Assessment.

**Table 2:** Global hemodynamic variables recorded in the three different time points during execution of the study protocol (n = 15)

	**T**_**BL1**_	**T**_**MX**_	**T**_**BL2**_
Heart rate, beats per minute	95 (4.3)	97 (4.4)	98 (4.4)
Systolic blood pressure, mm Hg	113 (4.6)	94 (4.0)^a^	111 (3.8)^a^
Diastolic blood pressure, mm Hg	52 (4.9)	49 (4.8)^a^	57 (4.9)^a^
Mean arterial blood pressure, mm Hg	75 (3.0)	61 (2.9)^a^	71 (2.3)^a^
Central venous pressure, mm Hg	12 (4.0)	9 (5.0)^a^	10 (6.0)
Cardiac index, n = 6, L/min per m^2^	4.1 (0.4)	3.8 (0.5)	3.9 (0.4)
Stroke volume, n = 6, mL	78 (15)	66 (14)	77 (12)

Data are presented as mean (standard error). ^a^*P* <0.05 versus previous time point (linear model for repeated measurements). Time points are defined as before nitroglycerin infusion (T_BL1_), at the maximum dose of nitroglycerin (T_MX_), and 30 minutes after cessation of nitroglycerin (T_BL2_). Cardiac index and stroke volume were measured in six patients.

**Figure 2:**
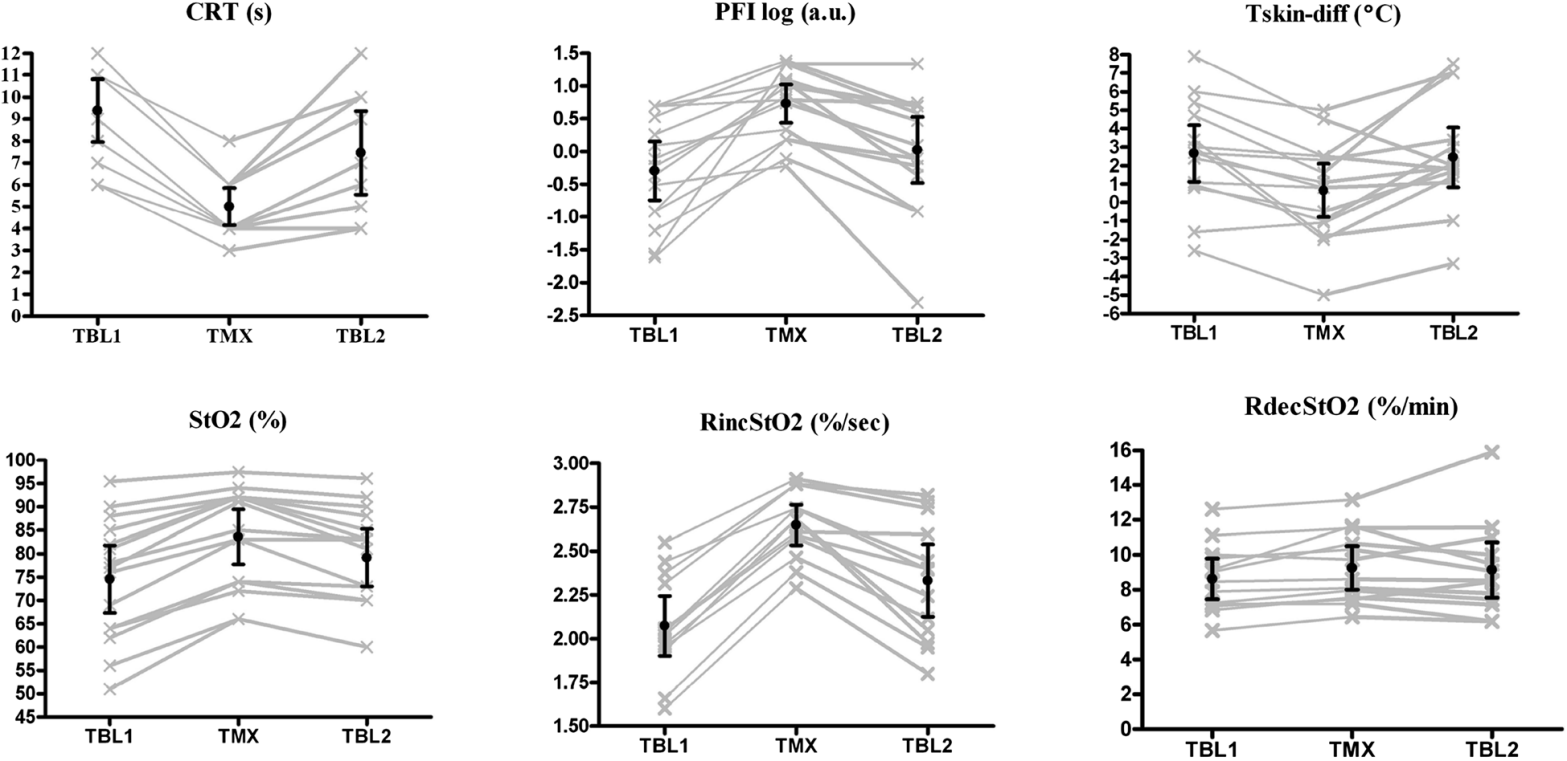
**Temporal behavior of peripheral circulation parameters (CRT, Tskin-diff, and PI) and StO**_**2**_**-derived variables (StO**_**2**_**, RincStO**_**2**_**, and RdecStO**_**2**_**) during study protocol.** Time points are defined as before nitroglycerin infusion (T_BL1_), at the maximum dose of nitroglycerin (T_MX_), and 30 minutes after cessation of nitroglycerin (T_BL2_). CRT, capillary refill time (seconds); PIlog, log of perfusion index (percentage); RdecStO_2_, rate of peripheral tissue oxygenation desaturation during arterial occlusion (percentage per minute); RincStO_2_, rate of peripheral tissue oxygenation recovery after arterial occlusion (percentage per second); StO_2_, peripheral tissue oxygenation (percentage); Tskin-diff, forearm-to-fingertip skin-temperature gradient (degrees Celsius). Lines represent individual values for each patient. Bars are mean ± 95% confidence interval (CI).

Baseline parameters of peripheral perfusion and the effects of nitroglycerin on these parameters are shown in Table 3. Two patients did not tolerate the 3-minute vascular occlusion test and therefore neither RincStO_2_ nor RdecStO_2_ is reported for these patients. Improvement in peripheral perfusion was reached in all patients. All 15 patients responded with more than 50% of CRT and PI of greater than 1.4% at the maximum dose of nitroglycerin, and nine patients responded additionally with Tskin-diff of less than -2. Twelve patients required a low dose of nitroglycerin (of less than 8 mg/hour) to normalize the peripheral perfusion parameters. We did not find differences in CRT, PI, and Tskin-diff at baseline between patients requiring low doses and patients requiring high doses. Therefore, all patients included shared abnormalities in peripheral perfusion before nitroglycerin infusion. Figure 2 shows the time course of peripheral perfusion parameters for each patient during nitroglycerin infusion at T_BL1_, T_MX_, and T_BL2_. Nitroglycerin infusion resulted in significant changes in CRT, Tskin-diff, and peripheral PI toward normal compared with baseline values: 51% (50% to 44%), 85% (30% to 112%), and 178% (105% to 295%), respectively. Similarly, we observed a significant increase in StO_2_ and RincStO_2_ values but not in RdecStO_2_ at T_MX_. All parameters returned to baseline values after cessation of nitroglycerin infusion.

**Table 3:** Peripheral perfusion parameters recorded in the three different time points during execution of the study protocol (n = 15)

	**T**_**BL1**_	**T**_**MX**_	**T**_**BL2**_
Capillary refill time, seconds	9.4 (0.6)	4.8 (0.3)^a^	7.1 (0.8)^a^
Tskin-diff, degrees Celsius	3.3 (0.7)	0.7 (0.6)^a^	1.8 (0.6)^a^
PI.log, percentage	-0.5 (0.2)	0.7 (0.1)^a^	0.2 (0.1)^a^
StO_2_, percentage	75 (3.4)	84 (2.7)^a^	79 (2.8)
Tissue hemoglobine index, arbitrary units	11.1 (1.3)	13.2 (1.4)^a^	11.6 (1.2)^a^
RincStO_2_, n = 13, percentage per second	1.9 (0.08)	2.8 (0.05)^a^	2.4 (0.09)^a^
RdecStO_2_, n = 13, percentage per minute	8.6 (0.5)	9.2 (0.6)	9.14 (0.7)

Data are presented as mean (standard error). ^a^*P* <0.05: previous time point (linear model for repeated measurements). Time points are defined as before nitroglycerin infusion (T_BL1_), at the maximum dose of nitroglycerin (T_MX_), and 30 minutes after cessation of nitroglycerin (T_BL2_). Rate of tissue oxygen saturation increase after arterial occlusion (RincStO_2_) and rate of tissue oxygen saturation deoxygenation during arterial occlusion (RdecStO_2_) were collected from 13 patients. PI, perfusion index; Tskin-diff, forearm-to-fingertip skin-temperature gradient.

Table 4 shows estimates from multiple regression analysis with respective confidence intervals predicting changes in CRT, Tskin-diff, peripheral PI, and StO_2_ for a given nitroglycerin dose. The effect of changes in nitroglycerin dose had a significant effect on CRT, Tskin-diff, and peripheral PI but not on StO_2_. When StO_2_ baseline values before nitroglycerin infusion were taken into account, the effect of changes in nitroglycerin dose becomes clearly significant. The magnitude of changes in StO_2_ was more prominent at lower StO_2_ values (Figure 2). Compared with baseline, patients with StO_2_ of less than 75% at T_BL1_ had a bigger response than patients with StO_2_ of greater than 75%: 10% (9.0% to 11.1%) versus 7% (4.7% to 10.5%) (*P* <0.05).

**Table 4:** Estimation of the effect of nitroglycerin dose on all parameters of peripheral perfusion

	**Estimate**	**95% CI**	***P *** **value**
Constant	8.9	4.60, 13.10	0.001
Capillary refill time, seconds	-0.91	-1.10, -0.50	0.001
Tskin-diff, degrees Celsius	0.35	0.09, 0.61	0.008
Perfusion index, percentage	1.2	0.55, 1.85	0.001
StO_2_, percentage	-0.02	-0.07, 0.03	0.42
StO_2_, percentage, corrected for baseline StO_2_	0.30	0.14, 0.47	0.001

CI, confidence interval; StO_2_, tissue oxygen saturation; Tskin-diff, forearm-to-fingertip skin temperature gradient.

## Discussion

We have demonstrated that intravenous infusion of nitroglycerin improves peripheral perfusion and oxygenation in shock patients with persisting abnormal peripheral circulation following initial resuscitation to global hemodynamic endpoints. Although nitroglycerin is generally used for its cardio-circulatory effects, its precise application in septic or non-septic circulatory shock continues to be debated and investigated. The use of vasodilators in shock was introduced into the clinic in the early 1960s as an additional therapeutic option for circulatory shock with or without cardiac dysfunction to counteract peripheral vasoconstriction [1,2,28]. However, the concept of using vasodilator therapy to target microcirculatory flow in critically ill patients originated in the 1990s with clinical trials of different types of vasodilators (prostacyclin and N-acetylcysteine) targeting splanchnic perfusion as assessed by gastric tonometry [29-32]. These studies demonstrated an improvement in gastric perfusion with vasodilator administration suggesting successful microcirculatory recruitment. More recently, with the advent of video microscopy techniques allowing direct visualization of the (sublingual) microcirculation, some studies have evaluated short-term infusions of nitroglycerin in septic or non-septic shock and demonstrated significant improvements in capillary perfusion [10-12]. In a randomized controlled trial in 70 patients with septic shock, Boerma and colleagues [11] failed to show significant differences in the evolution of the sublingual microcirculation between the control and nitroglycerin groups. Although this study precluded the effectiveness of nitroglycerin in the sublingual microvascular flow, the fixed dose of nitroglycerin used (2 mg/hour) was able to significantly increase skin blood flow as measured by central-to-toe temperature gradient in the treatment group. In addition to that finding, the authors reported lower Sequential Organ Failure Assessment (SOFA) scores in patients who received nitroglycerin compared with the placebo group. In our study, a dose of 2 mg/hour was not sufficient to improve peripheral perfusion in almost 80% of the patients, indicating that the nitroglycerin dose aiming to normalize abnormal peripheral perfusion should be individualized. In addition, since its dose-dependent effect can be easily predicted (Table 4) and the parameters of abnormal peripheral circulation can be easily obtained at the bedside, the use of nitroglycerin to correct an abnormal peripheral circulation can be easily implemented in clinical practice.

The persistent abnormalities in peripheral circulation have been shown to predict a poor outcome in critically ill patients in terms of mortality and multiple organ dysfunction [13-15,18,20,33]. To what extent peripheral perfusion parameters result in abnormal vital organ dysfunction causing these reported increases in morbidity and mortality is currently speculative. Nevertheless, the true clinical usefulness of peripheral perfusion monitoring can be supported only if it can be used to guide therapy to change outcome. Studies have shown that resuscitation procedures aiming at supporting global perfusion fail to normalize peripheral perfusion [34]. Therefore, more specific direct interventions to correct abnormalities in peripheral perfusion, such as a vasodilator, would be a first step to meet this resuscitation goal. The rationale of vasodilator therapy is based on the concept that blood flow in the peripheral circulation is regulated by changes in perfusion pressure, which is determined by intravascular pressure gradient and vessel radius of arterial peripheral circulation. Although the vessel radius has an important effect on flow (fourth power of the radius), the flow occurs only if there is a difference of pressure [35]. Microcirculatory perfusion pressure is, therefore, the net result of precapillary inflow pressure minus venular outflow pressure. From this physiologic perspective, a series of clinical studies have assessed the effect of vasodilators as potential adjunctive therapy to recruit microvascular perfusion in circulatory shock [10-12,29-32,36].

Nitroglycerin has some attributes that favor its use to recruit the microcirculation in critically ill patients. First, nitroglycerin has a quick onset of action (2 to 5 minutes) with a half-life elimination of 1 to 3 minutes. Second, nitroglycerin has specific hemodynamic effects in the venous capacitance vessels resulting in pressure gradient increase in the microvasculature and thus in microvascular blood flow. In this study, improvements in the clinical parameters of peripheral circulation were obtained in all patients. CRT improved in parallel with changes in Tskin-diff and peripheral PI, suggesting that the improvement in cutaneous circulation was likely the result of an increase in cutaneous blood flow. As the major role of cutaneous circulation is thermoregulation, blood flow to the skin typically exceeds metabolic requirements. Therefore, the high blood flow relative to its oxygen demand makes the skin an appropriate organ to detect variations in peripheral blood flow.

Another interesting finding in our study was that nitroglycerin infusion led to a significant improvement in peripheral tissue oxygenation. In addition, the magnitude of changes in StO_2_ was more pronounced in patients who had lower StO_2_ values (less than 75%). This finding may be explained by the shift of blood volume from arterial to venous compartment because of predominant venous dilation with a subsequent increase in the oxyhemoglobin levels within the volume of the vascular bed in the catchment area of the near infrared spectroscopy (NIRS) probe. Significant gain in StO_2_ is, therefore, observed in conditions in which the functional microcirculatory reserve is poor, such as those seen in abnormal peripheral circulation. In these conditions, a low initial StO_2_ value yields a greater percentage change. Alternatively, we found parallel changes in StO_2_ reoxygenation rate but not in the StO_2_ deoxygenation rate. Unlike the StO_2_ deoxygenation rate during arterial occlusion in which venous outflow and arterial inflow are blocked, StO_2_ recovery after the vascular occlusion reflects the sudden increase of arterial inflow during the hyperemic response. Infusing a vasodilator will result in a larger flow-mediated dilation following reactive hyperemia and therefore in a higher rate of reoxygenation. This pattern of changes in tissue oxygenation parameters is consistent with previous reports, including some of our own group, showing that StO_2_-derived parameters are influenced by peripheral vasoregulation [37-40]. Given that oxygen demand remained unchanged as reflected by constant StO_2_ deoxygenation rate in our patients, any increase in flow would result in an increase in microcirculatory hemoglobin saturation levels. Thereby, the noticeable changes in StO_2_ and StO_2_ recovery rate in our patients provide evidence that nitroglycerin markedly improves tissue oxygenation.

Although this study makes novel observations, a limitation to our study is the small number of patients. Despite this sample size, our patients shared the same baseline common abnormality in peripheral perfusion, and our study was designed to allow every patient to serve as his or her own control, thereby minimizing bias. Hence, the evident significant improvement and worsening in the clinical parameters of peripheral circulation in all patients during and after nitroglycerin infusion strengthen our findings. Another important point to mention is that the effect of nitroglycerin on skin and muscle perfusion as measured in our patients cannot be extrapolated to other organs, such as gastrointestinal tract, liver, or kidneys. However, the accumulating evidence from the literature supports the efficacy of vasodilator therapy in improving microcirculatory perfusion in these different vascular beds. We speculate, therefore, that nitroglycerin infusion in our patients had the same beneficial effect as seen in the peripheral tissues. Finally, our study supports the hypothesis that nitroglycerin dose should be individualized and shows that non-invasive and rapidly available measures of peripheral circulation at the bedside are available to predict its response and monitor the effect to reach an effective dose (Table 4). However, an important next step before the introduction of monitoring peripheral circulation and its treatment with individualized dose of nitroglycerin should be a randomized clinical trial showing clinical benefit for critically ill patients with persistent abnormal peripheral perfusion.

## Conclusions

We demonstrated with this study that stepwise dose of intravenous infusion of nitroglycerin reverses clinical abnormalities of peripheral circulation in patients with circulatory shock. In addition, we showed that the easy and reliable clinical parameters of peripheral perfusion can be an effective monitoring approach at the bedside to titrate the beneficial effects of nitroglycerin on peripheral circulation in individual patients with circulatory shock following initial resuscitation.

## Key messages

• Stepwise dose of intravenous infusion of nitroglycerin reverses clinical abnormalities of peripheral circulation in patients with circulatory shock.

• Nitroglycerin infusion response in some patients was observed with the dose higher than the conventional dose of 2 mg/hour.

• The easy and reliable clinical parameters of peripheral perfusion can be an effective monitoring approach at the bedside to titrate the beneficial effects of nitroglycerin on microcirculation in individual patients with circulatory shock following initial resuscitation.

## Abbreviations

CRT: capillary refill time; ICU: intensive care unit; PI: perfusion index; RdecStO_2_: rate of tissue oxygen saturation deoxygenation during arterial occlusion; RincStO_2_: rate of tissue oxygen saturation increase after arterial occlusion; StO_2_: tissue oxygen saturation; T_BL1_: time point at baseline before nitroglycerin infusion; T_BL2_: time point at 30 minutes after cessation of nitroglycerin infusion; T_MX_: time point at the maximum dose of nitroglycerin; Tskin-diff: forearm-to-fingertip skin-temperature gradient.

## Competing interests

The authors declare that they have no competing interests.

## Authors’ contributions

AL was involved with the conception and design of the work; acquisition, analysis, and interpretation of data; and drafting and revising the work critically for important intellectual content. JB was involved with the conception and design of the work; analysis and interpretation of data; and revising the work for important intellectual content. MEvG carried out the experiments and the data acquisition. EK and TJ helped to carry out the experiments. JvB participated in its design and coordination and helped to draft the manuscript. All authors read and approved the final manuscript.
